# Aerobic Exercise Training Improves Renal Injury in Spontaneously Hypertensive Rats by Increasing Renalase Expression in Medulla

**DOI:** 10.3389/fcvm.2022.922705

**Published:** 2022-07-11

**Authors:** Minghao Luo, Shuyuan Cao, Dingyi Lv, Longlin He, Zhou He, Lingang Li, Yongjian Li, Suxin Luo, Qing Chang

**Affiliations:** ^1^The Affiliated Rehabilitation Hospital of Chongqing Medical University, Chongqing, China; ^2^Department of Cardiology, The First Affiliated Hospital of Chongqing Medical University Chongqing, China; ^3^The College of Exercise Medicine, Chongqing Medical University, Chongqing, China

**Keywords:** SHR, exercise, renalase, kidney, HK-2

## Abstract

We aimed to examine the effects of aerobic exercise training on renal function in spontaneously hypertensive rats (SHR) and elucidate their possible mechanisms. Adult male SHR and age-matched Wistar-Kyoto rats (WKY) were divided into four groups: WKY sedentary group, SHR sedentary group, low-intensity training group, and medium-intensity training group. Using molecular and biochemical approaches, we investigated the effects of 14-week training on renalase (RNLS) protein levels, renal function, and apoptosis and oxidative stress modulators in kidney tissues. *In vitro*, angiotensin II (Ang II)-induced human kidney proximal epithelial cells (HK-2) were treated with RNLS, and changes in apoptosis and oxidative stress levels were observed. Our results show that moderate training improved renal function decline in SHR. In addition, aerobic exercise therapy significantly increased levels of RNLS in the renal medulla of SHR. We observed *in vitro* that RNLS significantly inhibited the increase of Ang II-inducedapoptosis and oxidative stress levels in HK-2. In conclusion, aerobic exercise training effectively improved renal function in SHR by promoting RNLS expression in the renal medulla. These results explain the possible mechanism in which exercise improves renal injury in hypertensive patients and suggest RNLS as a novel therapy for kidney injury patients.

## Introduction

Hypertension is a major threat to global public health, inducing coronary artery disease, heart attack, and stroke, as well as chronic kidney disease (CKD) and end-stage renal disease (ESRD) (1). According to the American Kidney Disease Data System, hypertensive renal damage is the second most common cause of ESRD (8.7%) (2). Data on dialysis patients with renal failure revealed that hypertension accounted for 17.9% of the total number of patients undergoing treatment, and this proportion shows an increasing trend (3). Therefore, effective control of hypertension and intervention for hypertensive kidney damage are urgent problems that need to be solved (4, 5).

Physical exercise is a natural remedy for many diseases and can lead to benign changes in most physiological systems, and it plays an important role in the prevention and treatment of chronic diseases (6, 7). Scheduled exercise reduces the risk of cardiovascular and all-cause death in patients with hypertension (8–12). Exercise includes aerobic exercise, impedance, and stretching (6, 13). Moderate aerobic exercise can lower blood pressure and has a protective effect on the cardiovascular system (11, 12). A meta-analysis suggested that aerobic exercise is a potential non-pharmacological treatment for improving blood pressure in patients with essential hypertension (14). Ruegsegger et al. reported that low-to-moderate aerobic exercise training could reverse hypertension (13). Exercise intensity is determined by maximum oxygen uptake (VO_2_max), and there exists a relationship between running speed and maximum oxygen uptake (15–17).

There are two free radical (FR) defense systems in the human body: an enzymatic defense system involving superoxide dismutase (SOD), glutathione peroxidase (GSH-Px), catalase (CAT), and glutathione reductase (GR), and a non-enzymatic defense system involving vitamin C, vitamin E, and glutathione (GSH). NADPH oxidases (NOXs) is the key enzymes involved in electron transfer in the cell membrane and is one of the main sources of reactive oxygen species (ROS). The body keeps a dynamic balance between the generation and the removal of FR (18–20). When the level of lipid peroxidation exceeds the body's antioxidant capacity, such as malondialdehyde (MDA), it induces oxidative stress and directly causes biofilm injury, the degeneration of intracellular proteins, leading to cell death, apoptosis, tissue damage, and disease (21–23). Besides, both excessive endoplasmic reticulum stress and oxidative stress may contribute to renal apoptosis. Previous studies have shown strong links between renal cell apoptosis and oxidative stress in the kidney lesion, and under the condition of the injury, the functional cell transition from a normal morphology to an apoptotic phenotype could be activated (24–26).

In the current study, exercise training was found to significantly prevent activated mitochondria-dependent apoptotic pathways observed in sedentary hypertension, as evidenced by changes in hypertension-induced components of Bax, cytochrome c, Bcl-2, activated caspase-9, and activated caspase-3 levels after exercise training in heart (27). Furthermore, exercise training significantly reduced cytosolic levels of cytochrome c as well as cytosolic and nuclear apoptosis inducing factor (AIF) in soleus muscle of hypertensive animals (28). Besides, a previous study showed that physical exercise improved brain cortex and cerebellum through reduction of pro-apoptotic Bax/Bcl-2 proportion (29). These results suggest that regular exercise training provides protection against apoptosis by altering various apoptosis regulatory proteins and by influencing mitochondrial-mediated apoptotic signaling mechanisms.

Renalase (RNLS), a flavin/adenine/dinucleotide-dependent amine oxidase, is secreted by the kidney into the blood and is expressed in the heart, liver, pancreas, and skeletal muscles (30, 31). RNLS expression is mainly related to renal function, perfusion, and catecholamine levels. Research suggests that RNLS is involved in catecholamine metabolism owing to its oxidase activity. Specific single nucleotide polymorphisms in RNLS are closely associated with an increased risk of essential hypertension, CKD, and cardiovascular disease (32). Previous studies have shown that serum RNLS levels are significantly associated with hypertension risk (33, 34). Currently, the molecular mechanism underlying the link between renalase and hypertensive renal damage has not been fully elucidated.

Tokinoya et al. found that RNLS concentration increased in the blood and skeletal muscle after 60 min of moderate-intensity aerobic exercise for 6 consecutive days (35). At present, there are no studies that explain the pathological or molecular mechanisms of RNLS in exercise therapy for hypertension and target organ damage. Here, we explored the effect of aerobic exercise intervention on RNLS expression in spontaneously hypertensive rats (SHR) for the first time and attempted to clarify the role of RNLS in hypertensive renal injury.

## Materials and Methods

### Materials and Antibodies

Primary antibodies against RNLS, NOX2, NOX4, Bax, Bcl-2, and cleaved caspase-3 were obtained from Proteintech (Chicago, IL, USA). Primary antibodies against β-actin and secondary antibodies were obtained from Bioss (Beijing, China). Human RNLS was obtained from Sigma-Aldrich (St. Louis, MO, USA). Unless otherwise indicated, all other chemicals used in this study were obtained from Sigma-Aldrich.

### Animals

Eight-week-old male SHR and age-matched normotensive Wistar-Kyoto (WKY) rats (Vital River Laboratory, Beijing, China) were used in the current study. The rats were maintained under standard conditions: temperature of 22 °C, humidity of 50%, and a 12-h light/dark cycle. All experimental conditions were specific pathogen-free. Rats were euthanized after the experiments. The kidney was excised to assess protein expression and localization, and serum was obtained for biochemical tests. All research procedures were approved by the Ethics Committee of Chongqing Medical University and complied with the regulations of the People's Republic of China on the Management of Laboratory Animals.

### Aerobic Exercise Training Treatment

Exercise protocols were performed on a treadmill (SA101C; SANS Biological Technology, Jiangsu, China) customized to research sports physiology and pathology characteristics of small experimental animals (220 V, 50 Hz; 8 trails for running; speed control 0–100 m/min with a resolution of 0.01 m/min) and connected to a treadmill-software for continuous speed monitoring (36).

The aerobic exercise training protocol used in this study followed the guidelines for animal exercise and training (37). After 1 week of adaptive exercise training (8 m/min speed for 1 h/day), the rats were randomly assigned to the following groups (8 rats per group): WKY sedentary group (WKY-S), SHR sedentary group (SHR-S), SHR low-intensity aerobic exercise training group (SHR-L) with a speed of 14 m/min, and SHR medium-intensity aerobic exercise training group (SHR-M) at a speed of 20 m/min (38). The exercise groups ran on a treadmill for 14 weeks-5 days a week, for 60 min each time, and the treadmill inclination of each exercise group was 0.

### Cell Culture and Treatment

Human renal proximal tubule epithelial cells (HK-2; ATCC, VA, USA) were cultured in DMEM medium (Gibco, NY, USA) supplemented with 10% FBS (Gibco) and 1% penicillin/streptomycin (Invitrogen, CA, USA) at 37 °C and 5% CO_2_. HK-2 cells were treated with 1 μM angiotensin II (Ang II; MedChemExpress, Shanghai, China) for 24 h to induce oxidative stress and establish a hypertensive renal injury model *in vitro*. To confirm the effect of RNLS on HK-2 cells, HK-2 cells were incubated with various concentrations of RNLS (10, 100, or 1000 ng/mL; Sigma-Aldrich) in the absence or presence of Ang II.

### Biochemical Analyses

Renal function and oxidative stress indicators in the serum were measured using reagent kits provided by the Nanjing Jiancheng Bioengineering Institute (Nanjing, China) (36). Serum creatinine (Scr) levels were measured using the sarcosine oxidase method. Blood urea nitrogen (BUN) levels were measured using the urease assay. Uric acid (UA) was measured using the uric acid enzymatic method. SOD activity was measured using the hydroxylamine method. GSH-Px activity was measured using a colorimetric method. MDA levels were measured using the thiobarbituric method.

### Blood Pressure Measurement

The mean blood pressure (MBP) was measured in conscious rats using a non-invasive tail-cuff system (Softron, Tokyo, Japan) (39). The rats were habituated to the tail-cuff procedure prior to the experiment. Before the measurements, rats were maintained in an incubator (37 °C) for 20 min. The blood pressure of each rat was determined by averaging three measurements.

### Western Blot

Protein expression was tested by western blotting as described previously (40). Briefly, cells and tissues were lysed on ice for 1 h in lysis buffer. Protein concentrations were measured by Bradford assay, and 40 μg of protein was used for western blotting. Proteins were separated by 10% sodium dodecyl sulfate-polyacrylamide gel electrophoresis and transferred onto polyvinylidene difluoride membranes, the membranes were blocked with 5% non-fat milk for 2 h at 37 °C and then incubated with primary antibodies overnight at 4 °C. Membranes were incubated with secondary antibodies conjugated with horseradish peroxidase at 37 °C for 2 h. Bands were detected using chemiluminescence detection reagent (Biosharp, Beijing, China).

### Immunofluorescence

Frozen sections of renal tissue were fixed with 4 % methanol for 20 min at room temperature and permeabilized with Triton-X100 in PBS for 20 min. Sections were blocked with 5 % goat serum for 30 min, incubated with rabbit anti-RNLS antibody (1:100) overnight at 4 °C, and then incubated with fluorescence-labeled goat-anti-rabbit secondary antibody (Beyotime, Shanghai, China) at 37 °C for 1 h in the dark. Finally, the tissue was stained with DAPI for 5 min. A laser scanning microscope (Leica Microsystems, Germany) was used to acquire images to assess the fluorescence signal of RNLS.

### Statistical Analysis

All data are expressed as Box-whisker Plot. Comparisons between groups were performed using one-way analysis of variance followed by Bonferroni's *post-hoc* test when data followed a normal distribution or with Mann-Whitney U test when data did not. A value of *p* < 0.05 was considered statistically significant. GraphPad software (version 8.0) was used for the analysis.

## Results

### Aerobic Exercise Improved Blood Pressure, Renal Function, and Oxidative Stress in SHR

**Figure 1:**
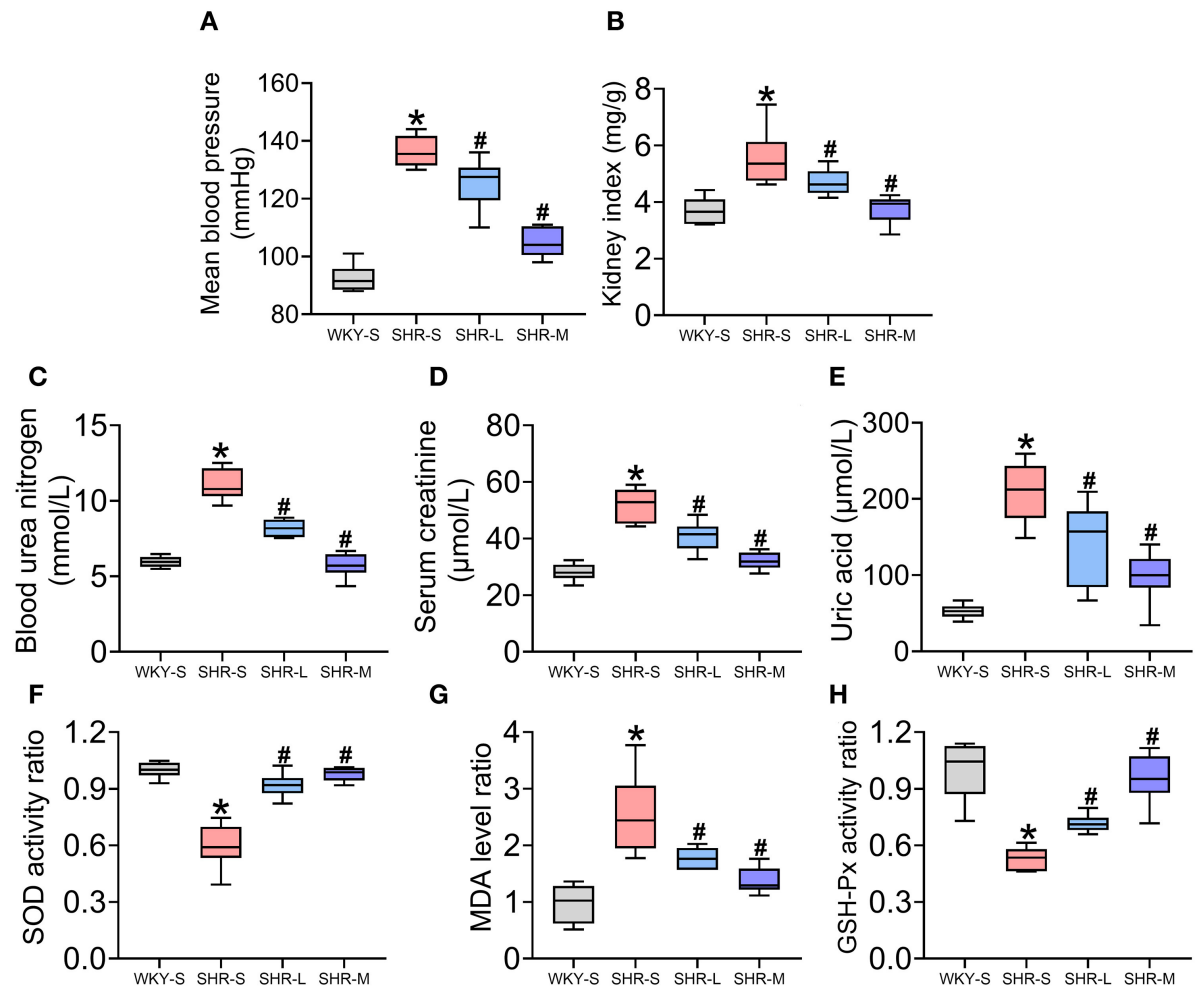
Effect of aerobic exercise training on mean blood pressure (MBP), renal function, and oxidative stress in spontaneously hypertensive rats (SHR). **(A)** MBP **(B)** kidney index **(C)** blood urea nitrogen **(D)** creatinine **(E)** uric acid **(F)** superoxide dismutase activity **(G)** malondialdehyde levels, and **(H)** glutathione peroxidase activity in serum were measured in all groups. Data are presented as Box-whisker Plot. ^*^P < 0.05 (WKY-S vs. SHR-S), ^#^*P* < 0.05 (SHR-L, SHR-M vs. SHR-S).

The role of aerobic exercise on MBP (Figure 1A) and renal function (kidney index, Scr, BUN, and UA) (Figures 1B–E) in hypertension was determined in SHR and WKY rats. MBP, kidney index, BUN, Scr, and UA were higher in SHR-S than in WKY-S (*P* < 0.05). Compared to SHR-S, in the SHR-L and SHR-M groups, MBP, kidney index, BUN, Scr, and UA decreased (*P* < 0.05).

To examine the effect of aerobic exercise on oxidative stress in SHR, SOD and GSH-Px activities (enzymatic defense system against free radicals) and MDA levels (indicator of lipid peroxidation) in serum were measured (Figures 1F–H). SOD and GSH-Px activities in SHR-S were significantly lower than those in WKY-S (*P* < 0.05) and significantly higher in SHR-L and SHR-M than in SHR-S (*P* < 0.05). Additionally, the MDA level in SHR-S was significantly higher than that in WKY-S and significantly lower in SHR-L and SHR-M than in SHR-S.

### Aerobic Exercise Improved Oxidative Stress and Apoptosis of Kidney in SHR

**Figure 2:**
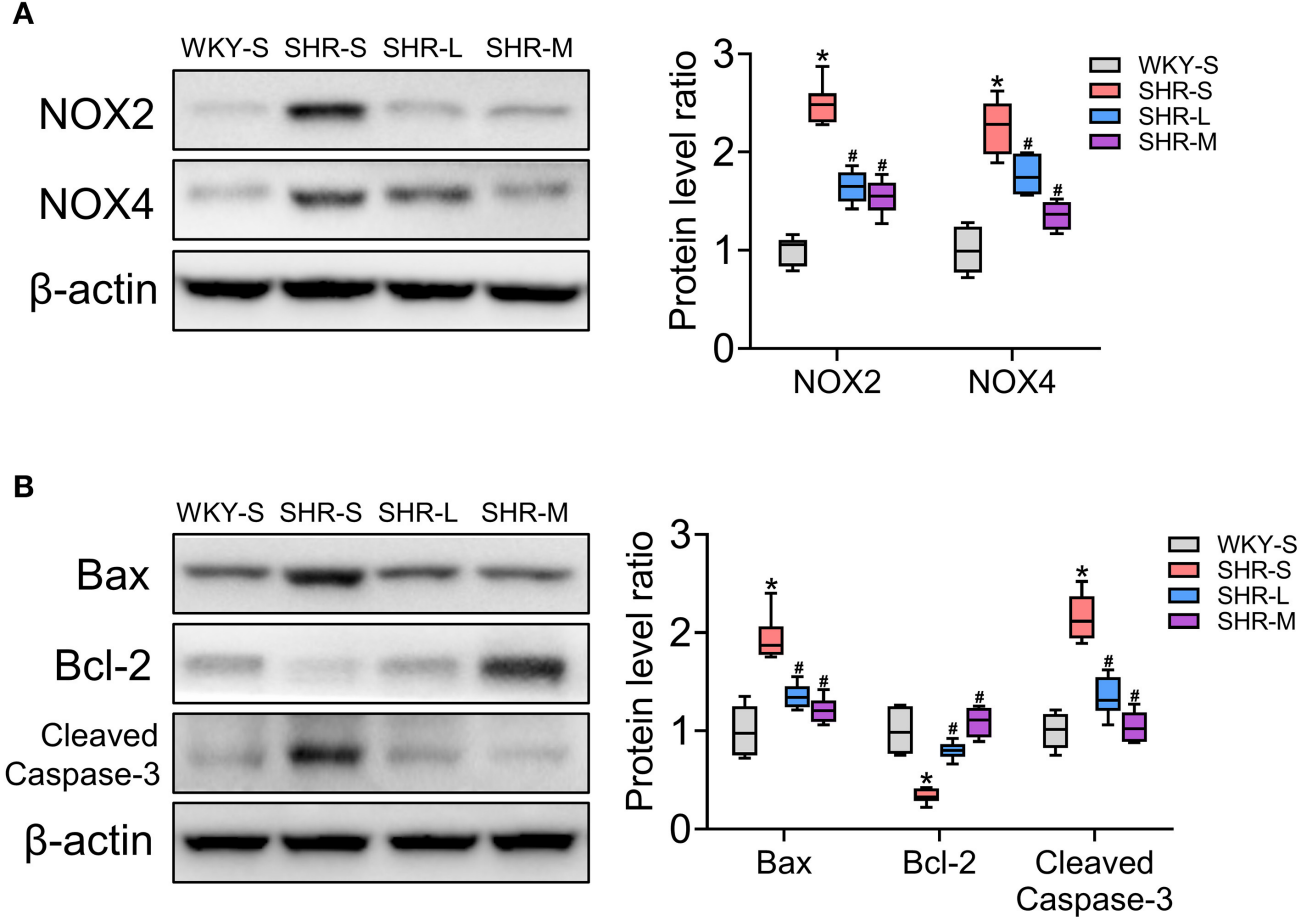
Effects of aerobic exercise training on **(A)** NOX2 and NOX4, and **(B)** Bax, Bcl-2, and cleaved-caspase-3 protein expression in kidney of SHR. Data are presented as Box-whisker Plot. *P < 0.05 (WKY-S vs. SHR-S), ^#^*P* < 0.05 (SHR-L, SHR-M vs. SHR-S).

NADPH oxidase 2/4 (NOX2/4) is a key enzyme involved in electron transfer in the cell membrane and is one of the main sources of reactive oxygen species (41, 42). To determine the level of oxidative stress, we measured the levels of NOX2 and NOX4 in the kidneys using western blot. We also explored the effect of exercise on apoptosis. To assess the influence of aerobic exercise training on apoptosis, the expression of pro-apoptotic proteins (Bax and cleaved caspase-3) and anti-apoptotic protein (Bcl-2) were examined in the studied groups (43). As shown in Figure 2, compared with the WKY-S group, oxidative stress (NOX2 and NOX4) and apoptosis (Bax, Bcl-2, cleaved caspase-3) in the kidney were markedly increased in the SHR-S group (*P* < 0.05). However, the oxidative stress and apoptotic indices were significantly lower in SHR-L and SHR-M groups than those in the SHR-S group (*P*< 0.05). These results showed that aerobic exercise attenuated oxidative stress and apoptosis in the kidneys of SHR.

### Effect of Aerobic Exercise on RNLS Expression in the Kidney of SHR

**Figure 3:**
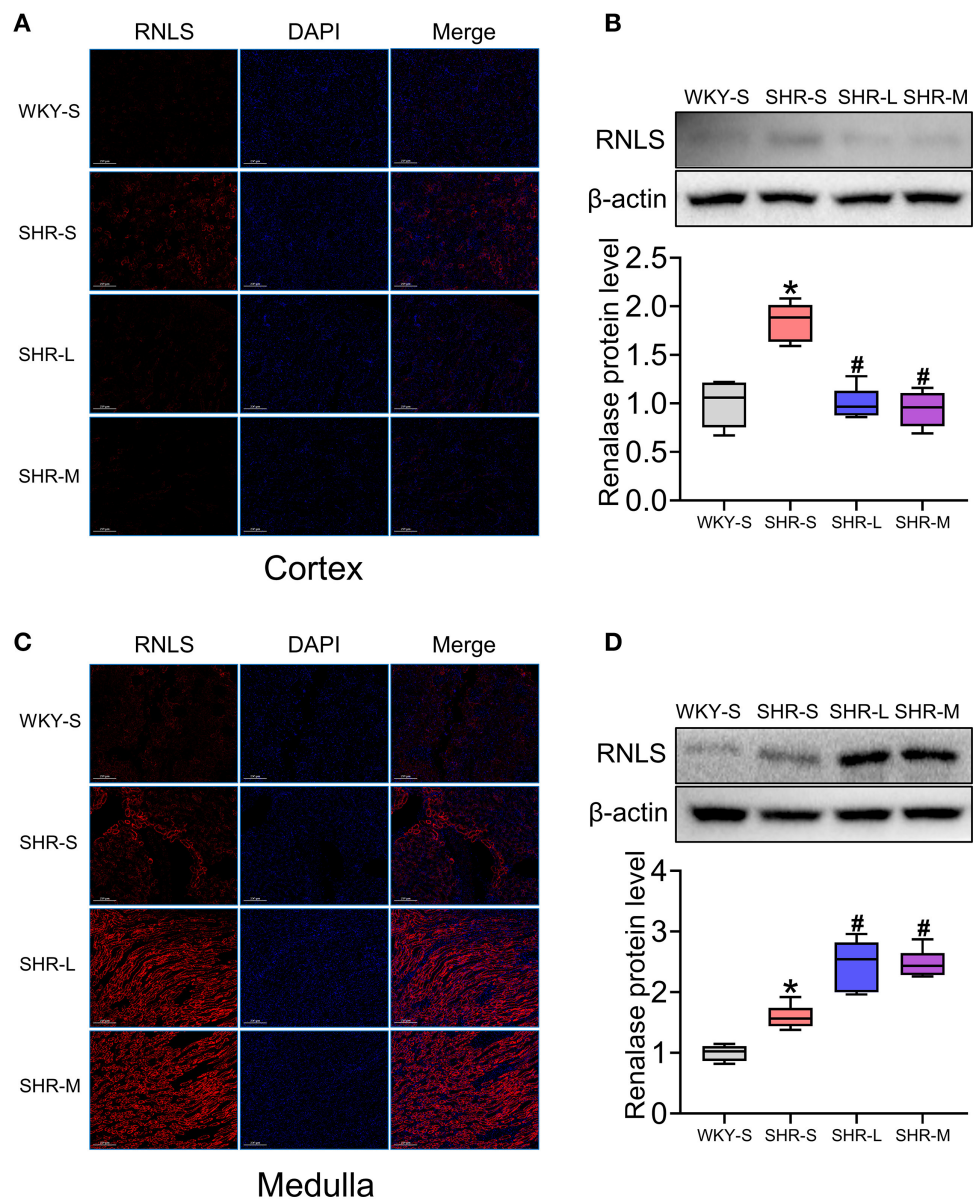
Effect of aerobic exercise training on RNLS protein expression in kidney of SHR. RNLS expression of renal cortex and medulla were determined using immunofluorescence **(A,C)** and western blot **(B,D)**. Data are presented as Box-whisker Plot. *P < 0.05 (WKY-S vs. SHR-S), ^#^*P* < 0.05 (SHR-L, SHR-M vs. SHR-S).

To determine the effect of aerobic exercise on RNLS expression in the kidneys of SHR, we tested RNLS expression in the renal cortex and medulla using immunofluorescence (Figures 3A,C) and western blot (Figures 3B,D). The results suggested that RNLS expression was significantly increased in the renal cortex and medulla of SHR (*P* < 0.05). However, the data indicated that aerobic exercise significantly inhibited RNLS expression in the renal cortex while significantly increased RNLS expression in the renal medulla. There were significant differences between the SHR-L and SHR-M groups and the SHR-S group (*P* < 0.05).

### RNLS Attenuated Ang II-Induced Oxidative Stress and Apoptosis in HK-2 Cells

**Figure 4:**
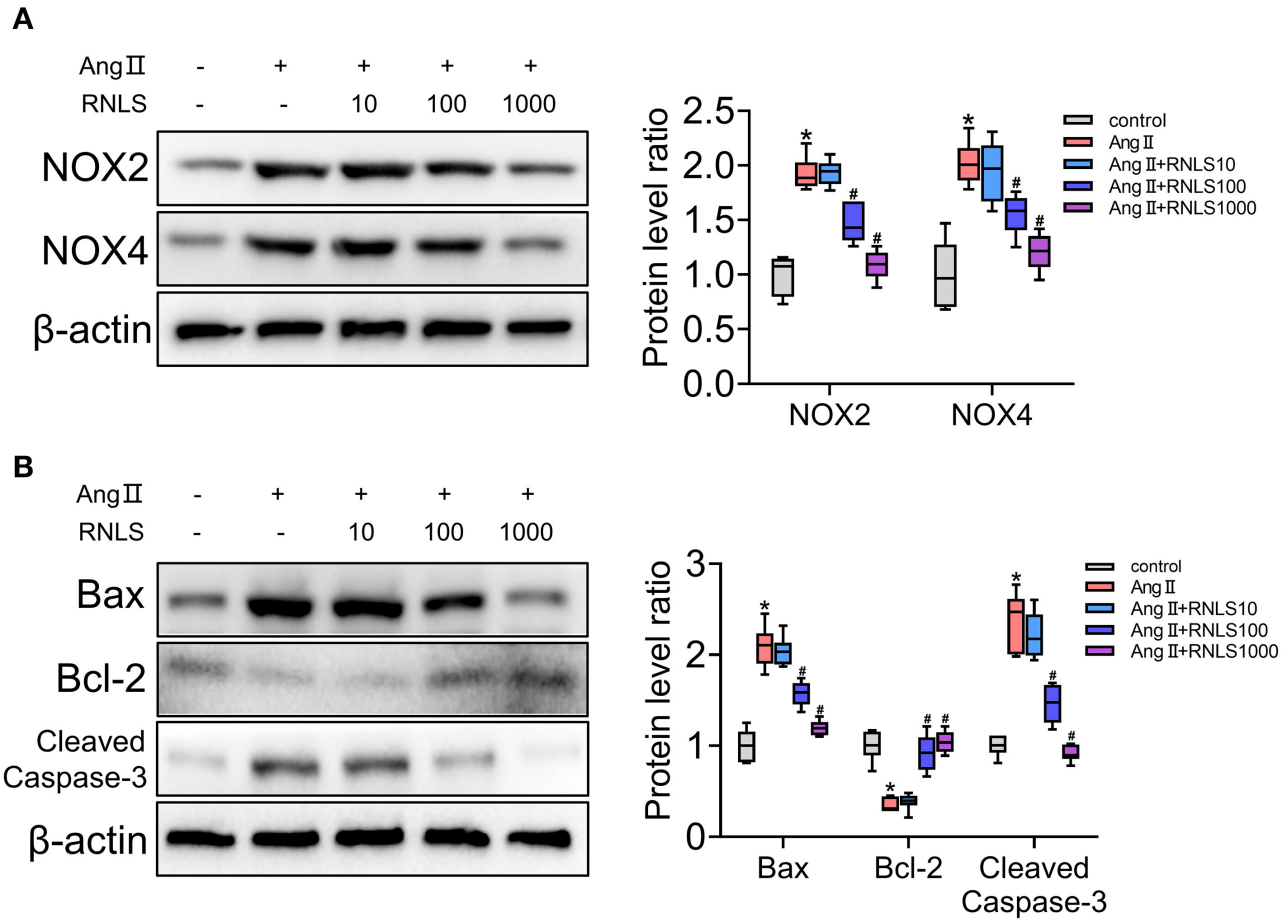
Effect of RNLS on angiotensin II (Ang II)-induced changes in HK-2 cells. **(A)** NOX2, NOX4 **(B)** Bax, Bcl-2, and cleaved-caspase-3 protein expression in HK-2 cells were detected using western blot. HK-2 cells were incubated with various concentrations of RNLS (10, 100, or 1000 ng/mL) in the absence or presence of Ang II (1 μM). Data are presented as Box-whisker Plot. ^*^P < 0.05 (Control vs. Ang II), ^#^*P* < 0.05 (Ang II+RNLS vs. Ang II).

To further verify and test the effect of RNLS on hypertensive renal injury, we conducted experiments using HK-2 cells. HK-2 cells were incubated with various concentrations of RNLS (10, 100, or 1000 ng/mL) in the absence or presence of Ang II (1 μM). Activation of oxidative stress (Figure 4A) and apoptosis (Figure 4B) were observed in HK-2 cells in response to Ang II. RNLS treatment significantly blocked the increase in NOX2, NOX4, Bax, and cleaved-caspase-3 expression and the decrease in Bcl-2 expression induced by Ang II in HK-2 cells (*P* < 0.05).

## Discussion

In the present study, we provided evidence that aerobic exercise training improves hypertensive kidney injury in rats. In addition, this improvement may be related to the increased expression of RNLS in the renal medulla. We observed, for the first time, the effect of exercise on RNLS expression in the kidney and the ameliorative action of RNLS on hypertensive renal injury.

Scheduled exercise in hypertensive patients reduces the risk of cardiovascular death and all-cause death (10). Therefore, in order to lower blood pressure in hypertensive patients, a total of 30–60 min of moderate-intensity exercise (such as walking, jogging, cycling, swimming, etc.) is recommended for 4–7 days a week, in addition to daily activities (6). Exercises can be aerobic, impedance, or stretching. Aerobic exercise was found to be more effective for symptom improvement in patients with hypertension. Therefore, we focused on aerobic exercise training in this study.

The intensity of exercise for benign regulation of blood pressure must vary from person to person. In clinical studies, the maximum heart rate during exercise is commonly used to evaluate the exercise intensity (13). Strictly speaking, exercise speed is measured and calculated through a typical progressive exercise test to measure the VO_2_ max, which utilizes the principles developed in rats (37, 38, 44). To determine maximal exercise capacity, rats underwent a progressive exercise test using an incremental speed protocol of 5 m/min every 3 min, with no grade until exhaustion. However, with extension of the exercise training cycle, the maximum oxygen intake and aerobic velocity in some SHR exhibited dynamic changes. We also referenced the treadmill speed based on the Guidelines for Animal Exercise and Training Protocols for Cardiovascular Studies, in which treadmill speed corresponding to different exercise intensities was introduced. In this study, low and moderate exercise intensities for SHR were set according to different running speeds. We consider that 40 m/min is 100% of the maximal aerobic speed (speed at VO_2_ max). Therefore, 14 m/min is 30–40% of maximal aerobic speed and 20 m/min is 45–55% of maximal aerobic speed, corresponding to low-intensity and moderate-intensity aerobic exercise running speed, respectively.

This study did not directly measure the content of ROS in kidney but observed the activity of SOD, GSH-Px, and MDA level, and the proteins expression of NOX2 and NOX4, which indirectly proved that exercise of appropriate intensity had a positive effect on the oxidative stress level in SHR. Our results revealed that low- and medium-intensity training could significantly improve oxidative stress. Most interestingly, we found that exercise reversed hypertension-induced elevation in the Bax/Bcl-2 ratio and activated caspase-3, supporting the protective effects of continuous aerobic exercise against increased apoptosis.

Studies have found that RNLS is likely to play an important role in the development of hypertension (45). Serum RNLS levels were higher in hypertensive subjects than in normal subjects and positively correlated with blood pressure (33). However, RNLS expression was significantly reduced in renal biopsy specimens from patients with hypertension compared to those from patients with normal blood pressure. At present, the molecular mechanism underlying the link between RNLS and hypertensive kidney injury has not been fully elucidated, but several hypotheses have been proposed (46). First, recombinant RNLS exerts a powerful and rapid antihypertensive effect in rodents, which is thought to be mediated by the degradation of circulating catecholamines, reducing cardiac contractility and heart rate, thus regulating kidney perfusion. Second, RNLS reduces sympathetic activity by metabolizing circulating catecholamines (such as dopamine, adrenaline, and norepinephrine), which indirectly causes hemodynamic changes and is conducive to improving renal dysfunction and inflammation induced by sympathetic abnormalities. Third, as a new target for cardiovascular disease and CKD, renalase have shown potential anti-inflammation and antioxidant stress effects in cardiac and renal protection.

The effect of exercise on renal RNLS remains controversial. Bozena et al. found that in the kidney, RNLS protein levels decreased after training, whereas mRNA levels increased (47). Katsuyuki et al. reported that RNLS protein levels in the kidney decreased after moderate-intensity exercise and high-intensity exercise training (35, 48). Meanwhile, these studies suggested that RNLS was significantly elevated in the skeletal muscle after exercise training. Interestingly, we investigated changes in RNLS expression in two major parts of the kidney and obtained different results. Notably, our research suggested that aerobic exercise significantly inhibited RNLS expression in the renal cortex, while significantly increased RNLS expression in the renal medulla. In addition, western blot and immunofluorescence results indicated that the RNLS fluorescence intensity in the renal medulla was higher than that in the renal cortex. The mechanism of distinct exercise effects on RNLS in different renal parts needs to be further studied. The results of this study in the renal cortex were consistent with those of previous studies; however, the medulla showed completely opposite results. Previous studies that did not mention which part of the kidney was evaluated do not conflict with our results. We hypothesized that the observed effect of aerobic exercise on renal injury in SHR, including oxidative stress and apoptosis, is closely related to the significant increase in renal medullary RNLS. On the one hand, RNLS is an amine oxidase that regulates blood flow by metabolizing catecholamines and improves the injury caused by insufficient perfusion. In contrast, previous studies have shown that RNLS directly affects the function of renal tubular cells (49, 50).

Renal medulla locates in the deep layer of the renal cortex, occupying about two-thirds of the kidney. The main components of renal medulla are renal tubules, including the ascending thick limbs of Henle's loop (TAL), distal tubules (DT) and medulla collecting tubules. Renal tubules have the function of material transport, including reabsorption and secretion. Meanwhile, renal tubule epithelial cells can regulate renal function by secreting various factors (49). Our data suggested that the level of RNLS in DT and TAL was significantly increased after exercise, while the level of RNLS in renal tubular epithelial cells of cortex was lower than that of DT. Previous studies did not pay attention to the differences in renal tubule epithelial cells between the cortex and the medulla, which is also the innovation point of this study.

Studies have shown that RNLS inhibit oxidative stress and apoptosis *in vitro* (50–52). RNLS protects renal epithelial cells in cisplatin-induced Acute Kidney Injury by promoting mitochondrial dynamics and inhibiting ROS production in a Sirt3-dependent manner (50). RNLS deficiency in cardiac tissue exacerbates myocardial damage after myocardial ischemia and reperfusion, and exogenous RNLS protein protects against this damage by reducing cell necrosis and apoptosis (53). Pretreatment with RNLS protected against contrast-induced nephropathy via anti-oxidation, anti-apoptosis, and anti-inflammation mechanisms (54). In this study, we showed that RNLS improved Ang II-induced oxidative stress and apoptosis in HK-2 cells.

This study revealed that continuous aerobic exercise had positive effect on oxidative stress and renal dysfunction in SHR. Both low intensity and medium intensity could improve the renal dysfunction and the oxidative stress of SHR, while our previous research found adverse effects of HICT on blood pressure and oxidative stress (36). A key finding was observed in this research: Both low intensity and medium intensity could significantly improve the renal dysfuntion of SHR, while medium intensity showed a better effect. This also provides certain experimental evidences for us to consider the intervention method on renal dysfunction by aerobic exercise in the future, that is, the most appropriate exercise intensity is around 50% VO_2_max.

In summary, the results of this study clearly demonstrated that aerobic exercise significantly ameliorated renal dysfunction in SHR by increasing RNLS levels in the renal medulla. Our study provides new insights into how aerobic exercise can ameliorate hypertension complications and suggests that RNLS may be a valuable option for treatment of hypertensive kidney injury.

## Data Availability Statement

The original contributions presented in the study are included in the article/supplementary material, further inquiries can be directed to the corresponding author.

## Ethics Statement

The animal study was reviewed and approved by Ethics Committee of Chongqing Medical University.

## Author Contributions

Conceptualization, validation, data curation: ML and SC. Methodology: LH, ZH, LL, and YL. Software: ML and DL. Formal analysis: SC. Investigation and supervision: QC and SL. Resources and funding acquisition: QC and SC. Writing—original draft preparation, writing—review and editing, and visualization: ML. Project administration: QC. All authors have read and agreed to the published version of the manuscript.

## Funding

This project was supported by Scientific and Technological Research Program of Chongqing Municipal Education Commission (Grant No. KJQN202100443).

## Conflict of Interest

The authors declare that the research was conducted in the absence of any commercial or financial relationships that could be construed as a potential conflict of interest.

## Publisher's Note

All claims expressed in this article are solely those of the authors and do not necessarily represent those of their affiliated organizations, or those of the publisher, the editors and the reviewers. Any product that may be evaluated in this article, or claim that may be made by its manufacturer, is not guaranteed or endorsed by the publisher.
